# Food Antioxidants and Their Anti-Inflammatory Properties: A Potential Role in Cardiovascular Diseases and Cancer Prevention

**DOI:** 10.3390/diseases4030028

**Published:** 2016-08-01

**Authors:** Keith Griffiths, Bharat B. Aggarwal, Ram B. Singh, Harpal S. Buttar, Douglas Wilson, Fabien De Meester

**Affiliations:** 1 Emeritus Professor of Cancer Research, University of Wales College of Medicine, Laurel Cottage, Castleton, Cardiff CF3 2UR, UK; profkgriffiths@aol.com; 2 Anti-inflammation Research Institute, San Diego, CA 92093, USA; bbaggarwal@gmail.com; 3 Halberg Hospital and Research Institute, Civil Lines, Moradabad, UP 244001, India; rbs@tsimtsoum.net; 4 Department of Pathology & Laboratory Medicine, Faculty of Medicine, University of Ottawa, Ottawa, ON K1H 8M5 , Canada; hsbuttar@bell.net; 5 School Medicine Pharmacy and Health, Durham University, Durham TS17 6BH, UK; 6 The Tsim Tsoum Institute, Krakow 31-534, Poland; fdm@tsimtsoum.net

**Keywords:** flavonoids, carotenoids, vitamins, pro-oxidants, stress, inflammation, transcription factors, microbiome, spices

## Abstract

Mediterranean-style diets caused a significant decline in cardiovascular diseases (CVDs) in early landmark studies. The effect of a traditional Mediterranean diet on lipoprotein oxidation showed that there was a significant reduction in oxidative stress in the intervention group (Mediterranean diet + Virgin Olive Oil) compared to the low-fat diet group. Conversely, the increase in oxidative stress causing inflammation is a unifying hypothesis for predisposing people to atherosclerosis, carcinogenesis, and osteoporosis. The impact of antioxidants and anti-inflammatory agents on cancer and cardiovascular disease, and the interventive mechanisms for the inhibition of proliferation, inflammation, invasion, metastasis, and activation of apoptosis were explored. Following the Great Oxygen Event some 2.3 billion years ago, organisms have needed antioxidants to survive. Natural products in food preservatives are preferable to synthetic compounds due to their lower volatility and stability and generally higher antioxidant potential. Free radicals, reactive oxygen species, antioxidants, pro-oxidants and inflammation are described with examples of free radical damage based on the hydroxyl, nitric oxide and superoxide radicals. Flavonoid antioxidants with 2- or 3-phenylchroman structures such as quercetin, kaempferol, myricetin, apigenin, and luteolin, constituents of fruits, vegetables, tea, and wine, which may reduce coronary disease and cancer, are described. The protective effect of flavonoids on the DNA damage caused by hydroxyl radicals through chelation is an important mechanism, though the converse may be possible, e.g., quercetin. The antioxidant properties of carotenoids, which are dietary natural pigments, have been studied in relation to breast cancer risk and an inverse association was found with plasma concentrations: higher levels mean lower risk. The manipulation of primary and secondary human metabolomes derived especially from existing or transformed gut microbiota was explored as a possible alternative to single-agent dietary interventions for cancer and cardiovascular disease. Sustained oxidative stress leading to inflammation and thence to possibly to cancer and cardiovascular disease is described for spices and herbs, using curcumin as an example of an intervention, based on activation of transcription factors which suggest that oxidative stress, chronic inflammation, and cancer are closely linked.

## 1. Introduction

Recent results from the PREDIMED studies indicate that Mediterranean-style diets can cause a significant decline in cardiovascular diseases (CVDs) and cancer [1,2,3]. The beneficial effects of Mediterranean-style diets may be because of the increased intake of fruits, vegetables, nuts, fish, poultry and olive oil with very little red meat; these are foods which have a low glycaemic index [1,2,3]. The majority of these foods possess increased content of polyphenolic flavonoids, carotenoids, omega-3 fatty acids, antioxidants, vitamins and minerals as well as essential and non-essential amino acids [4,5]. Increased consumption of nuts has been demonstrated to cause a significant decline in all-cause mortality [6], whereas a greater intake of fresh fruits was associated with significant decline in CVDs in China [7]. Mediterranean-style diets have also been demonstrated to cause a significant decline in CVDs in earlier landmark studies [8,9,10,11]. The effect of a traditional Mediterranean diet on lipoprotein oxidation showed that there was a significant reduction in oxidative stress in the intervention group (Mediterranean diet + Virgin Olive Oil) compared to the low-fat diet group [12]. Conversely, the increase in oxidative stress causing inflammation is a unifying hypothesis for predisposing atherosclerosis, carcinogenesis, and osteoporosis [13,14,15] and for type 2 diabetes, the latter not forming part of this review. Clearly, intervention, experimental, and epidemiological studies, particularly prospective studies, may enhance our understanding of how flavonoids, carotenoids and vitamins may regulate gene function through signalling pathways and have an impact on cancer incidence/survival, or risk of CVD. In an earlier publication, Griffiths demonstrated an important role of oestrogens, phyto-oestrogens, in the pathogenesis of prostatic disease [16]. This review examines the role of oxidative stress and inflammation in the pathogenesis of CVDs and cancer.

## 2. Food Preservation, Oxidative Stress and Antioxidant Balance

Antioxidants are additives that preserve food from ‘farm to plate’, and prevent deterioration in storage and processing. Vegetables and fruit are often stored and sealed in a low oxygen atmosphere at low temperatures in the dark, but despite this intervention, oxidation reactions still take place during storage. The antioxidants are an important category of food preservatives, natural or synthetic, designed to prevent food from spoiling through oxidation, thus reducing loss of nutrients, and maintaining texture, colour pigments, taste, freshness, functionality, and aroma. They also have a major physiological role in inter- and intra-cellular signalling mechanisms and metabolic processes in plant and animal life. They also comprise antimicrobial or antifungal phytoalexins (Ahuja et al. [17]), synthesized de novo following pathogen invasion, particularly isoflavonoids, in defence against pathogen invasion. Consequently, their physiological effects and metabolic fate and impact on cardiovascular diseases (CVDs) and prostate cancer [16] are discussed. To achieve potential success, intervention studies may provide much needed mechanistic and clinical information on, for example, the inhibition of proliferation, inflammation, invasion, metastasis, and the activation of apoptosis (Kuntz et al. [18] and Murtaza et al. [19]), rather than the more difficult epidemiological investigations which are thwarted with difficulties (Wang [20]).

To assist the general reader, this introduction is somewhat didactic in first describing the characteristics of free radicals, oxidants, and antioxidants, and in subsequent sections how these impact health and disease, notably CVDs and cancer. Excellent reviews, e.g., on free radicals, antioxidants, and functional foods; on stress, vascular inflammation and antioxidants and cardiovascular disease; and on phyto-oestrogens (antioxidant properties) and cancer have been published by Lobo et al. [21], Siti et al. [13] and Hwang and Choi [22], respectively. In an evolutionary context, perhaps 50 million years before the Great Oxygen Event some 2.3 billion years ago [23], atmospheric oxygen became increasingly present, though variable, in the once-anaerobic world and defence mechanisms were evolved to combat its toxicity and adapt to the developing world up to the falling level of 21% of today. ‘Circadian’ periods were also increasing (Table 1 in Williams [24]) due to tidal friction and there was an increase in the diversity of life forms with eukaryotes in post-Precambrian times who had longer lifespans and were able to develop their energy needs beyond a single day-length. The distribution of oxygen occurrence in the atmosphere, oceans, and land became relatively stable and free radicals, oxidants, antioxidants were essential for the survival of life forms, and as such are important today from the metabolic, wholesome food, and human medicine perspectives. Natural products are preferable to synthetic compounds as food additives or preservatives due to their lower volatility and stability, their relatively higher antioxidant potential, and though not infallible, their safety and benefit, as well as consumer preference.

## 3. Free Radicals, Oxidants, and Reductants

### 3.1. Free Radicals

The term ‘radical’ has been known since 1790 [25] but its terminology has varied [26] until the work of Gomberg [27,28], who is recognized as the father of ‘radical’ chemistry (albeit limited in understanding because molecular theory, then, was still in its infancy). One technique for the identification of free radicals is electron-spin resonance, first observed by Zavoisky [29,30], the foundations of which have been published by Eaton [31]. Hertzberg [32], the Nobel Prize Laureate in chemistry (1971), described free radicals as any transient (chemically unstable) species (atom, molecule, or ion). Today, a radical, often referred to perhaps unnecessarily as a ‘free’ radical, is an atom or groups of atoms that have one or more unpaired electrons, and as a consequence are highly reactive and may inflict cellular/genetic damage. Some notable radicals loosely listed according to oxygen, nitrogen, sulphur and carbon, respectively, include: hydroxyl (HO^*^), superoxide anion (O_2_^−*^), hydroperoxyl (HOO^*^), and alkyloxyl (ROO^*^) radicals; nitric oxide (NO^*^) and other oxides of nitrogen; thiyls (RS^*^), disulphide anions (RSSR^−*^); and carbonate (CO_3_^−*^), whose reactivity, as judged from rate constants of these oxidants with substrates, comes close to that of chemical diffusion, e.g., hydroxyl radicals may be a proximal factor (nm) of cell damage [33].

There is a paradox here, because radicals are also beneficial and function as inter-cellular and intra-cellular signalling systems. Their daily production is about four million according to Ayala et al. [33] and so must be balanced. For instance, oxygen radicals also are essential to life when generated in the mitochondria in the electron transport chain, and they are also involved in the enzymatic reactions essential to intermediary metabolic processes of life. When free oxygen or nitrogen radicals are produced in the organism, which are commonly termed reactive oxygen species (ROS), they can become dominant and are not adequately controlled due to the low availability of antioxidants, and a condition of oxidative stress occurs which adversely affects cells and tissue in the body, thereby causing damage to the cardiovascular system and giving rise to heart attack, stroke and cancer-related conditions [34,35].

### 3.2. Oxidants

An oxidant such as hydrogen peroxide (H_2_O_2_) is a chemical that removes or scavenges electrons from a reactant.

The term oxidant or oxidation comes from the element oxygen (Greek: acid-former from the Greek roots οξυς (*oxys*) meaning ‘acid’ or ‘sharp’ and γεινομαι (*geinomai*) meaning ‘engender’), named by Lavoisier in ca. 1775, who described strong oxyacids as entities containing central atoms in high oxidation states such as nitrogen and sulphur, which lose electrons during reactions with oxygen. The hypothesis that it was the oxidation of food which provided animal heat and motion was ascribed by Mayer as published by Tyndall in 1879 [36]. In biology, quite apart from ‘free radicals’, some of which are listed above, there are two-electron oxidants and numerous other peroxides such as alkyl or aryl hydro-ROOH or endo-ROOR peroxides, various carbonyls, hypo-halogen acids, peroxynitrate (ONOOH), etc., as published, for example, by Davies [37]. There are also many excellent early reviews on this subject [38,39,40].

### 3.3. Reductants

In a redox chain reaction, one reactant is the reducing agent which is an electron donor, and the other is the oxidizing agent which is the electron recipient. A reductant is a substance that loses or donates an electron(s); as such, antioxidants are called reductants. Simply put, an oxidizing agent is a chemical species that removes electrons from another: it is one component of an oxidation-reduction, or redox, pair. Antioxidants, vide infra, are scavengers of certain free radicals.

## 4. Examples of Free Radical Damage

Only an overview of the free radical interactions is presented here. More comprehensive reviews may be found in the cited references [16,41,42,43].

### 4.1. Hydroxyl Radical (HO^*^)

Radiation damage in humans interacts with water with the formation of the hydroxyl radical [44], which, if generated in the vicinity of DNA, can interact with the pyrimidine and purine bases to form mutagens [45] such as 8-hydroxyguanine from the parent purine via oxidation of the 8-hyroxy adduct of guanine. This radical reacts with the heterocyclic moiety of the thymine and cytosine purines at C5 and C6 positions, resulting in the C5–OH and C6–OH adduct radicals, respectively [46], ultimately leading to the formation of the cytosine and thymine glycols, respectively, and impaired double-stranded DNA. The hydroxyl radical can also interact with the sugar moiety of DNA, leading to intermolecular cyclization and, in the case of purines, the production of 8,5′-cyclopurine-2′-deoxynucleosides with resultant breaks in DNA strands and base-free sites. It is estimated that this radical produces four million molecules per day, which are naturally obtained though the Fenton reaction [47] involving hydrogen peroxide and transition metals *M*_n_ (Co, Cu, Fe Ni, V), which can be recycled by the resultant oxidized form *M*_n+1_ by interaction with the superoxide anion (O_2_^−*^) [48] to form O_2_ [49]. Further details on metal-induced oxidative stress and human disease have been published by Jomova et al. [50].

### 4.2. Nitric Oxide Radical (NO^*^)

This radical, after interaction and conversion to other radicals, e.g., with the superoxide anion O_2_^−*^ to form peroxynitrite [51], interacts with protein and can cause cell damage. It also reacts with molecular oxygen and nitrogen to form nitrogen dioxide and dinitrogen trioxide; both of these are toxic oxidizing and nitrosating agents [16,41] and cuprous (Cu^++^) and ferrous (Fe^++^) ions exacerbate this toxicity which is ameliorated by ion uptake of transport proteins in plasma, specifically ferritin, tranferrin for Fe^++^ and ceruloplasmin for Cu^++^. Nitric oxide (NO) is now recognized as an important endothelium-derived relaxing factor responsible for vasodilatation. NO has been implicated in red blood cell haemoglobin (RBC Hb) vasodilation due to carriage by a cysteine (Cys) within the β-chain, the βCys93 of haemoglobin. This amino acid residue has been assigned a role in S-nitrosothiol (SNO)-based hypoxic vasodilation by RBCs which is potentially important when mutation results in myocardial ischemia under certain conditions [52].

### 4.3. Superoxide Anion Radical (O_2_^−*^)

According to the Nobel Laureate Mechnikov [53], phagocytes such as neutrophils, monocytes, macrophages, mast cells, and dendritic cells are mobilized through chemotaxis to the site of bacterial infection and harm mediated through their surface receptors, and the ‘phagocytized’ bacteria are killed through a process implicating O_2_^−*^ [54]. These authors [54] over 40 years ago ‘hypothesized’ that the enzyme system responsible for the respiratory burst of phagocytosis provides the source of O_2_^−*^ and that this system is associated with the cell membrane. In as much as the same membrane forms the boundaries of the phagocytic vacuole, the mechanism for converting molecular oxygen to superoxide, thence to peroxide, would be located between the captured bacteria and the cytoplasm of the cell.

## 5. Naturally Occurring Flavonoids, Carotenoids and Pro-Oxidants

### 5.1. Flavonoids

For the purposes of this report, the structures of flavonoids have been comprehensively documented by Griffiths et al. [16,55], particularly in relation to the pathogenesis of prostatic disease, and nutrition and cancer. Pietta [56], Nijveldt et al. [57] and Nimse and Pal [43] have more recently reviewed the mechanisms of action of natural antioxidants. Only compound names will be used to describe these benzo-γ-pyran derivatives herein, perhaps better known as 2- or 3-phenylchroman structures for flavonoids or isoflavonoids, respectively. These compounds consist of quercetin, kaempferol, myricetin, apigenin, and luteolin, and are found in fruits, vegetables, tea, wine and other plant-derived beverages and may reduce the incidence of coronary disease in elderly humans [58]. Using an evidence-based approach for the risk of CVD, a meta-analysis of 14 observational cohorts was performed by Wang et al. [59]. Summary findings for intakes of flavonoids, flavonoids, flavonols, anthocyanidins, proanthocyanidins, flavones, flavanones and flavan-3-ols showed a significantly decreased risk of CVDs with an inverse association with the risk of CVD when comparing the highest and lowest categories of flavonoid intake. A similar association was observed for flavonol intake and CVD risk. Sensitivity and subgroup analyses further supported this association. The summary relative risk (RR) for CVD for every 10 mg/day increment in flavonol intake was 0·95 (95% confidence interval (CI) was between 0·91 and 0·99), but a reduction of all-cause cancer risk could not be demonstrated [60] as cited by Griffiths et al. [55]. A later multicentre interview-based study by Petrick et al. [61] generally showed inconclusive results. Cases were diagnosed between 1993 and 1995, with either oesophageal adenocarcinoma (OEA), gastric cardia adenocarcinoma (GCA), oesophageal squamous cell carcinoma, and other gastric adenocarcinoma [61], and food frequency-matched controls were interviewed and cancer cases were followed up to the year 2000. Food frequency questionnaire responses were linked with the USDA Flavonoid Databases and the available literature for six flavonoid classes and lignans. However, anthocyanidins were associated with a decreased risk of mortality for GCA and modestly for OEA, but it is emphasized that CIs were wide.

**Figure 1:**
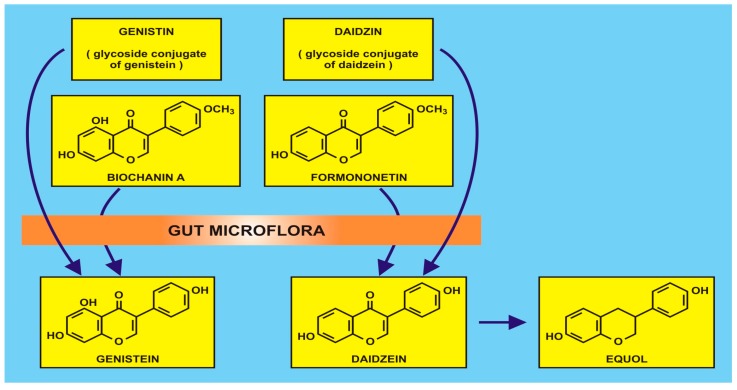
An example of isoflavonoid production by gut microflora (with the kind permission of David Griffiths, Castleton, Gwent).

Romagnolo and Selmin [62] have accumulated evidence on several cancer sites and found that food flavonoids exert protective effects against various types of tumours, including oral and pharyngeal, gastric, pancreatic, colorectal, hepatic, prostate, ovarian, endometrial, breast, and lung cancers (e.g., Turati et al. [63]). Even null effects have been published for the risk of gastric cancer in association with the intake of isoflavones [64] in women, and there appears to be an inconsistent association with some tea catechins (Carlson et al. [65]). Figure 1 shows some catechins isolated from green tea (q.v. also [55] (p. 41)) considered to be antioxidants. However, the problems with many studies are that flavonoid intake data are retrospective, lifestyle and dietary intake vary with time, the individual microbiome is not considered, agricultural production processes and growing conditions are often variable, and there are sociodemographic factors, etc., all of which may contribute to the variation in cancer incidence and survival, making flavonoid data difficult to interpret. As mentioned in the Introduction, intervention, experimental, and epidemiological studies, particularly prospective studies, may enhance our understanding of how flavonoids, carotenoids and vitamins may regulate gene function through signalling pathways and have an impact on cancer incidence/survival or risk of CVD. Therefore, such studies may provide useful mechanistic and clinical information, such as biomarkers of flavonoid intake and response.

**Figure 2:**
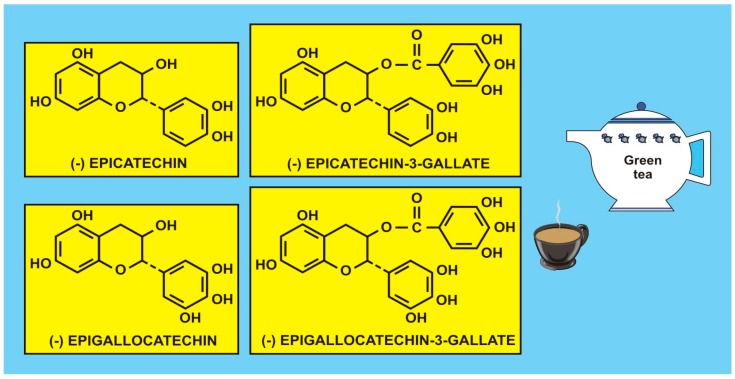
Flavonoid polyphenols, catechins, isolated from green tea (with the kind permission of David Griffiths, Castleton, Gwent).

Due to the aforementioned difficulties in dietary studies, attention is being focused on functional foods (rather than ‘superfoods’, often a misnomer), defined as natural or processed foods that contain known or unknown biologically-active compounds. In this present review, flavonoids in the foods are adjudged to be clinically effective in non-toxic amounts that provide proven and documented health benefits for the prevention, management, or treatment of chronic diseases, viz. primary and secondary human metabolomes derived especially from existing or transformed gut microbiota. The commensal gut microbiota have long been known to be involved in isoflavonoid metabolism (q.v. Griffiths et al. ([55] (p. 28)), of which there are many species of anaerobes, which may be manipulated by dietary intake. Methodological approaches can involve 16S ribosomal RNA sequence analysis to identify the diversity of bacterial genera, e.g., *Actinobacteria*, *Bacteroidetes* and *Firmicutes* combined with solid-phase microextraction-gas chromatography-mass spectroscopy of faecal specimens for quantification of short-chain fatty acids. Such acids, e.g., acetic, butyric and propionic acids, provide an epidemiological dimension to health and disease when investigating nutrition and cancer. Examples of metabolism by gut microflora are the isoflavonoids such as genistin and diadzin (and their methylated derivatives, biochanin A and formononetin) which are converted to genistein and daidzein, as shown in Figure 2.

Short-term dietary intervention studies using plant- or animal-based and other selected diets may lead to beneficial changes in cancer, cardiovascular-related or other diseases. In this respect, an acute human intervention pharmacokinetic trial with flavonoids to investigate absorption, metabolism and excretion from chocolate, orange juice and blackberries (COB) has been registered with Clinical Trials (website maintained by the National Library of Medicine at The National Institutes of Health, Bethesda, MD, USA), viz. Trials Registration Number, Clinical Trials. GOV (NCT01922869), in which the primary outcomes are flavonoid metabolites and putative functional single nucleotide polymorphisms (SNPs) within selected genes, and it will involve the genotyping of approximately 200 SNPs. Secondary measures are to determine the effects of gut microbiota variation on flavonoid metabolism by assessing gut microbiota using faecal bacterial phylogenetic analysis with PCR to amplify 16S rDNA genes. Another trial (NCT00677599), entitled ‘The FLAVO Trial: Dietary flavonoids and cardiovascular disease risk reduction in postmenopausal women with type 2 diabetes (FLAVO)’, by Curtis et al. [66] has been completed. This placebo-controlled trial was designed to determine whether a year-long intervention with flavonoids (found in cocoa and soy) is more effective in reducing the risk of CVD in postmenopausal women with type 2 diabetes, compared to standard therapy (statins). The outcome was that clinically relevant improvements in arterial stiffness were observed and that the one-year duration of the intervention was insufficient to produce potentially more meaningful results [66]. A one-year intervention with flavan-3-ols and isoflavones improved the biomarkers of CVD risk, highlighting the additional benefit of flavonoids with the standard drug therapy in managing CVD risk in postmenopausal type 2 diabetic patients [67].

Flavonoids have been reported to have a protective effect on the DNA damage caused by hydroxyl radicals [68], presumably through chelating metal ions, such as copper or iron. The flavonoids, complexed with the copper or iron, prevent the generation of ROS. However, some flavonoids such as quercetin may have either a protective or damaging effect of ROS (due to HO^*^, O^2−*,^ HOO^*^ upon DNA, depending on the concentration of chelating metal ions [69,70,71,72,73,74,75,76,77]).

### 5.2. Carotenoids

There have been a number of studies which suggest that dietary carotenoids may reduce breast cancer risk. Peto et al. [78] described the process of β-carotene’s cancer-protective effect as a process to scavenge free radicals. The early epidemiological work of Hirayama et al. [79] on various cancer types from 1966 to 1982 and the case-control dietary frequency questionnaire study of Chinese women used by Lee et al. [80] on breast cancer risk indicated the possibility that β-carotene was a protective factor for breast cancer and, perhaps, in the case of studies done by Hirayama et al. [79], for prostate cancer as well, with caution being drawn due to other dietary constituents such as green-yellow vegetables and soybean paste soup. To emphasize caution, the work of Boyle and Maisonneuve [81] directs attention to a Finnish study of β-carotene administration versus placebo (*n* = 23,000 Finnish male smokers) in which 138 cases of prostate cancer were found compared with 112 cases in the placebo group. However, the restraining effect of β-carotene on cellular damage has been reported by Krinsky [82]. In this context, a study by Singh et al. [83] of 1667 hospital-based patients in India, this cohort having a range of diagnosed medical conditions, compared with 202 controls, drew the following conclusions. Dietary consumption of antioxidant vitamins A, E, and C and β-carotene was lower in the majority of conditions compared to the controls. Plasma concentrations of vitamin C and β-carotene were significantly lower in all patient groups. Reduced vitamin E levels were noted in patients with CVDs and stroke, etc. Lipid peroxides, indicative of free radical damage, were significantly higher than in controls with most conditions, and greater overall in acute myocardial infarction, cancer, stroke patients. However, later studies of lung cancer by Gallicchio et al. [84] did not substantially support that β-carotene supplementation is associated with decreased risk of developing lung cancer, but methodological factors may be a potential cause of discord with prospective studies.

Eliassen et al. [85] carried out a pooled analysis of plasma carotenoids in breast cancer patients from eight cohort studies (3055 cases and 3956 matched controls) that represent nearly 80% of the world’s prospective published data and found statistically significant inverse associations with the risk of breast cancer for α-carotene, lutein + zeaxanthin, lycopene, and total carotenoids. For several carotenoids, associations were adjudged stronger for oestrogen receptor negative (ER (−)) than for oestrogen receptor positive (ER (+)) tumours. There was a suggestion that intakes of α-carotene, β-carotene, and lutein/zeaxanthin were inversely associated with the risk of ER- but not ER+ in breast cancer, but these must be treated with caution and an underlying mechanism must be sought to support/underpin these results [86]. There appears to be no significant reported adverse effects of carotenoids on breast cancer risk.

More recently, Eliassen et al. [87] conducted a nested case-control study of plasma carotenoids in patients from the Nurses’ Health Study at the Harvard School of Public Health and Brigham and Women’s Hospital in Boston, Massachusetts. Briefly, in 1989–1990 and 2000–2002, 32,826 and 18,743 (second sample) subjects, respectively, donated blood samples. In this study by Eliassen, between the first blood collection and June 2010, 2188 breast cancer cases were diagnosed (579 cases were diagnosed after the second collection) and matched with control subjects and data was analysed using conditional logistic regression adjusted for several breast cancer risk factors. Higher concentrations of α-carotene, β-carotene, lycopene, and total carotenoids were associated with 18%–28% statistically significant lower risks of breast cancer. Associations were apparent for total carotenoids measured ≥10 year before diagnosis as well as those <10 year before diagnosis. Importantly, plasma carotenoid concentrations were strongly inversely associated with breast cancer recurrence and death. These results strongly suggest that, over 20 years, the high level of carotenoids was associated with a lower breast cancer risk.

The suppression of tumour growth has been limited by the use of uncommon carotenoids such as crocins from saffron (Bolhassani [88]) because of their water-soluble glycosylated form, unlike the chemotherapeutic potential of lipophilic carotenoids limited by their occupancy of lipophilic compartments rather than their need to be water-soluble for scavenging free radicals, inhibition of angiogenesis, prevention of cell propagation, and apoptosis induction in breast, prostate and other cancers. Lycopene, a red pigment from tomatoes, can reach higher concentrations in prostatic tissue than some others and is chemotherapeutically more effective (Chen et al. [89]), though knowledge of signalling pathways involving carotenoids may provide better chemotherapeutic agents (Khuda-Bukhsh et al. [90]).

### 5.3. Pro-Oxidants

These are substances which induce oxidative stress either by generating reactive oxygen species or by reducing the effects of anti-oxidants through some action, either anti- or pro-oxidant, depending on the experimental conditions [91]. Vitamin C is an anti-oxidant when it reduces hydrogen peroxide, but is a pro-oxidant when it reduces metal ions such as iron to generate free radicals through the Fenton reaction [47], with the metal ion undergoing redox cycling. Study design issues may be the reason why Carr and Frei [92] could not uniquely answer the question ‘Does vitamin C act as a pro-oxidant under physiological conditions?’. This potential duality viz. anti- and pro-oxidant has been recognized for plant polyphenols [93,94,95,96,97,98,99] as reported by Azmi and Sarkar [100].

## 6. Spices, Aromatic Herbs, Antioxidants and Health

India is renowned for spices and medicinal plants, and turmeric, in particular, is used for wound healing, rheumatic disorders, gastrointestinal symptoms, etc. More recent studies have been focused on cancer prevention (e.g., Hackshaw-McGeagh et al. [101]) and anti-inflammatory (e.g., Gosh et al. [102]) conditions and treatment (e.g., Kumar et al. [103]). Curcumin (diferuloylmethane isolated from rhizomes of *Curcuma longa* (turmeric: ginger family *Zingiberaceae*)) was found to increase detoxifying enzymes, prevent DNA damage, improve DNA repair, and decrease mutations and tumour formation (q.v. reviews by Surh and Chun [104], Miriyala et al. [105], Krishnaswamy [106], Oyagbemi et al. [107], and Gerhauser [108]). It is useful to define herbs and spices given the wide spectrum of these natural and highly cultivated products found and grown in many other countries such as China, Malaysia, Pakistan, Bangladesh, Indonesia, and elsewhere [106].

A spice is a seed, fruit, root, rhizome, bark, resin, berry, bud, stigma, or vegetable substance primarily used for seasoning and provides sweet or savoury flavourings, colours or preserves food, or is a medicinal product, cosmetic or simply a vegetable. Spices include coriander, fennel, mustard, cinnamon, mace, clove, saffron, ginger, asafoetida, bay leaf oil, cinnamon, cloves, cumin, fenugreek, turmeric, poppy seed, pomegranate, red chili, sesame seed, and so forth. Many countries have spice mixtures, e.g., chaat masala (Pakistan and India). Herbs are parts of leafy green plants used for flavouring or to garnish culinary dishes. Aromatic herbs include thyme, sage, oregano, parsley, dill, marjoram, chives, rosemary, mint, lemon grass etc. Of course, spices and herbs are used as fresh, dry, or paste preparations. It has been reported that antioxidant capacity is still present during the processed end-product, as are the total phenolics, some being less degraded than others [109], for many herbs and spices in terms of total phenolic content. Spices or red wine are especially rich in polyphenols such as rosmarinic acid, and when added to meat or hamburgers [110] before cooking may exert a beneficial effect via a reduction of the formation or absorption from the gastrointestinal tract of malondialdehyde, a biologically active compound important in cytotoxic lipogenesis; elevated levels are associated with an increased risk of atherogenesis and cancer through mutagenic adducts from deoxyadenosine and deoxyguanosine in DNA and lipid peroxidation [111,112,113,114,115].

Rather than focusing on single-agent constituents of the diet, such as those total phenolics found in spices, it is perhaps more worthwhile to highlight the in vitro and in vivo evidence of potential spice benefits and to develop a hypothesis and potential biochemical mechanisms within a life-style framework. As a generalization, spices act as antioxidants, digestive stimulants, and hypolipidemics, and exhibit antibacterial, anti-inflammatory, antiviral, and anticarcinogenic activities as reported by Viuda-Martos et al. [116]. They are likely to be beneficial in a wide range of inflammatory diseases such as those described by Aggarwal et al. [117] and Prasad and Aggarwal [118]. Curcumin, for example, has been shown to have anti-inflammatory and antioxidant properties, and it displays high reactivity towards peroxyl radicals, and thus acts as a free radical scavenger. In this context, curcumin, extracted from turmeric, may serve as a chemotherapeutic agent and may have a preventative action against colon, skin, oral, and intestinal cancers [119]. A group belonging to of our co-authors (BA) (Sandur et al. [120]) demonstrated that curcumin (diferuloylmethane) exhibited antioxidant, anti-inflammatory, and proapoptotic properties, mediated through its pro-oxidant/antioxidant mechanisms. In this cited paper [120], tumour necrosis factor (TNF)-mediated nuclear factor kappa-light-chain-enhancer of activated B-cells (NF-κB) activation was inhibited by curcumin which was reversed by glutathione (GSH). Similarly, suppression of TNF-induced Akt (a serine/threonine kinase) activation by curcumin was also countered by GSH. Glutathione also countered the inhibitory effects of curcumin on TNF-induced NF-κB–regulated antiapoptotic (Bcl-2, Bcl-xL, IAP1), proliferative (cyclin D1), and proinflammatory (COX-2, iNOS, and MMP-9) gene products. Suppression of TNF-induced AP-1 activation by curcumin was also reversed by GSH. Also, the direct proapoptotic effects of curcumin were inhibited by GSH. Moreover, curcumin induced the production of reactive oxygen species and modulated intracellular GSH levels. Based on these results, Aggarwal’s group concluded that curcumin mediates its apoptotic and anti-inflammatory activities through modulation of the redox status of the cell.

Reuter et al. [121] have cited research detailing the mechanism by which sustained oxidative stress can lead to inflammation, thence further chronic conditions, e.g., cancer and CVDs. Oxidative stress can activate a variety of transcription factors including NF-κB, AP-1, p53 (the gene that codes for a protein that regulates the cell cycle and hence functions as a tumour suppressor), hypoxia-inducible factor 1-alpha (HIF-1α), peroxisome proliferator-activated receptor gamma (PPAR-γ), β-catenin/Wnt (regulates stem cell pluripotency and cell fate decisions during development), and Nrf2 (basic leucine zipper (bZIP) protein which regulates the expression of antioxidant proteins that protect against oxidative damage initiated by injury and inflammation.). Activation of these transcription factors can lead to the expression of over 500 different genes, including those for growth factors, inflammatory cytokines, chemokines, cell cycle regulatory molecules, and anti-inflammatory molecules. Evaluation of the published literature to date indicates that oxidative stress, chronic inflammation, and cancer are closely linked.

## 7. Conclusions

The goal of this work was to identify perspectives on food antioxidants and their potential for the prevention of cardiovascular diseases and cancer, and associated inflammatory diseases. A somewhat didactic approach was used to define terms, such as free radicals, reactive oxygen species, antioxidants and pro-oxidants and to illustrate their genesis. These terms were interwoven in the quest for the potential value of dietary intervention studies to reduce the impact of sustained oxidative stress leading to inflammation and thence to, possibly, cancer and cardiovascular disease, as described for flavonoid, carotenoid and pro-oxidant interventions. Similarly, the use of spices and herbs, using curcumin as an example of an intervention, based on the activation of transcription factors, suggests that oxidative stress, chronic inflammation, and cancer are closely linked. The manipulation of primary and secondary human metabolomes derived especially from existing or transformed gut microbiota was explored as a possible alternative to single-agent dietary interventions for cancer and cardiovascular disease. Hungin et al. [122] distinguish between probiotics, prebiotics and synbiotics, and the importance of gut microbiota for health through brain-gut-liver-immune interactions was the topic of a recent symposium in Miami, FL [123] Thus, natural dietary products provide evidence-based interventions through clinical and epidemiological studies, leading to the identification of transcription mechanisms that can ameliorate chronic stress and inflammation, as well as cancer and cardiovascular risk. The concept of dysbiosis needs further exploration.
